# Non-Invasive Brain Stimulation to Enhance Post-Stroke Recovery

**DOI:** 10.3389/fncir.2016.00056

**Published:** 2016-07-27

**Authors:** Nathalie Kubis

**Affiliations:** ^1^Service de Physiologie Clinique, AP-HP, Hôpital LariboisièreParis, France; ^2^Université Paris Diderot, Sorbonne Paris Cité, CART, INSERM U965Paris, France

**Keywords:** cerebral ischemia, stroke, rTMS, tDCS, plasticity

## Abstract

Brain plasticity after stroke remains poorly understood. Patients may improve spontaneously within the first 3 months and then more slowly in the coming year. The first day, decreased edema and reperfusion of the ischemic penumbra may possibly account for these phenomena, but the improvement during the next weeks suggests plasticity phenomena and cortical reorganization of the brain ischemic areas and of more remote areas. Indeed, the injured ischemic motor cortex has a reduced cortical excitability at the acute phase and a suspension of the topographic representation of affected muscles, whereas the contralateral motor cortex has an increased excitability and an enlarged somatomotor representation; furthermore, contralateral cortex exerts a transcallosal interhemispheric inhibition on the ischemic cortex. This results from the imbalance of the physiological reciprocal interhemispheric inhibition of each hemisphere on the other, contributing to worsening of neurological deficit. Cortical excitability is measurable through transcranial magnetic stimulation (TMS) and prognosis has been established according to the presence of motor evoked potentials (MEP) at the acute phase of stroke, which is predictive of better recovery. Conversely, the lack of response to early stimulation is associated with a poor functional outcome. Non-invasive stimulation techniques such as repetitive TMS (rTMS) or transcranial direct current stimulation (tDCS) have the potential to modulate brain cortical excitability with long lasting effects. In the setting of cerebrovascular disease, around 1000 stroke subjects have been included in placebo-controlled trials so far, most often with an objective of promoting motor recovery of the upper limb. High frequency repetitive stimulation (>3 Hz) rTMS, aiming to increase excitability of the ischemic cortex, or low frequency repetitive stimulation (≤1 Hz), aiming to reduce excitability of the contralateral homonymous cortex, or combined therapies, have shown various effects on the functional disability score and neurological scales of treated patients and on the duration of the treatment. We review here the patients’ characteristics and parameters of stimulation that could predict a good response, as well as safety issues. At last, we review what we have learnt from experimental studies and discuss potential directions to conduct future studies.

## Introduction

Stroke is the second leading cause of death, the second leading cause of dementia (Joray et al., 2009; Ovbiagele and Nguyen-Huynh, 2011; Roger et al., 2011) and the first cause of morbidity in industrialized countries. Reperfusion therapies such as thrombolysis using recombinant tissue plasminogen activator (Hacke et al., 2008), and more recently thrombectomy with a stent retriever, can rescue brain tissue of the penumbra (a rim of mild to moderate ischemic tissue around the core of the infarct), and improves the final neurological outcome (Fransen et al., 2014). Yet, it remains accessible to less than 5–10% of the population since the therapeutic window is restricted to 6 h (Wahlgren et al., 2016). In addition, the development of neuroprotective pharmacological treatments to limit the neuronal loss induced by ischemia proved disappointing when translating from experimental studies to clinical studies (Klein et al., 1999). The dogma, according to which, any brain injury is irreversible in adults and cannot be repaired has long prevailed both in medical schools and at the bedside. Yet, after a stroke, patients can improve spontaneously within the first 3 months (Maulden et al., 2005) and then more slowly in the following year. The first day, decreased oedema and partial reperfusion of the ischemic penumbra may possibly explain these phenomena, but the improvement of neurological deficit in the following weeks suggests plasticity phenomena and brain cortical reorganization (Chen et al., 2002). Restoring arm and hand skill after a stroke remains challenging, even though stroke rehabilitation programs have proven partial efficacy. Due to the worldwide increasing number of strokes predicted for 2030 (Béjot et al., 2016), and to the restricted number of centers able to provide reperfusion therapies in a limited therapeutic window, there is a need to develop new strategies that aim to enhance spontaneous cerebral plasticity. The complication comes from that, in stroke, post-lesional brain plasticity may be beneficial or “adaptive” or, detrimental or “maladaptive” and thus hamper neurological recovery.

The aim of this study is not to extensively review all the clinical studies published so far in the literature (for very complete reviews refer to Simonetta-Moreau (2014), dedicated to stroke, or Lefaucheur et al. (2014), about Non-Invasive Brain Stimulation [NIBS] in general neurology). The purpose of this review is rather to propose mechanisms from clinical and experimental data, and how up-coming clinical trials could be designed to better address these issues.

## Post-Stroke Brain Plasticity

### Definitions

Cortical plasticity is the capability of the cerebral cortex to modify its functional organization as a result of experience (Nudo, 2006). Consequently, all the changes that occur in brain organization after a repeated stimulus, refers to “plasticity”. Synaptic plasticity refers to the modification of the strength of synaptic transmission based on its firing history. All the plasticity forms coexist at a single synapse. Synaptic transmission can be either enhanced or depressed, and at various time scales: short-term synaptic and long-term synaptic plasticity have thus been described. Long term potentiation (LTP), is the most well-studied process involved in learning and memory: brain rewires and modifies its neural network to the formation of new memories. But plasticity has also been described after brain injury. The definition is purely descriptive and does not tell us much about the mechanisms that are involved: it underpins the capacity of the brain to change its functional and structural organization (histological and anatomical) in response to injury, to maximize the use of the remaining undamaged brain.

### The Excitability of the Neural Networks Located Near and Remote from the Injured Area Is Modified After Stroke

Since most patients recover at least partially, positive or adaptive plasticity occurs with less or more success. Plasticity mechanisms include activity-dependent rewiring and synapse strengthening. A sustained increase of glutamate release through brain derived nerve factor (BDNF) enhances synaptic activity (Carmichael, 2012). Animal models show that there would be a time-limited window of neuroplasticity following a stroke, during which the greatest gains in recovery occur (Murphy and Corbett, 2009). The challenge is to understand what are the mechanisms involved in post-stroke recovery to promote them optimally in each individual.

A further difficulty is that, at the same time, opposing effects take place. The peri-infarct area presents at the acute phase a decreased activity in relation with an upregulation of GABA mediated tonic inhibition (Clarkson et al., 2010). This has been interpreted as a neuroprotective mechanism to limit excitotoxicity and neuronal death. Blocking GABAergic activity during this 1-month period would enhance behavioral recovery. This decreased activity at the acute phase can be measured in the patient through transcranial magnetic stimulation (TMS). The principle developed more than 20 years ago consists of activating the cortex via a coil, inside which circulates an electric current of high intensity. The brief discharge of a few microseconds generates a magnetic field of 2–2.5 Tesla for a period of 0.3–1 ms. The magnetic current generated by the coil placed over the scalp, crosses the skull to the cortex. According to the Faraday principle, it induces an electric field in the cortex, which disappears beyond a depth of approximately 3 cm. This electric field then depolarizes neurons of the cortex beneath the coil, directly through their axon hillock or indirectly through depolarization of interneurons. Consequently, when TMS is applied over the motor cortex, a contralateral involuntary muscle contraction is elicited; the resulting motor evoked potential (MEP) is characterized by its amplitude, correlated to the number of neurons that have responded to the stimulation, and its latency, which measures the conduction time between stimulation and the onset of the MEP. Prognostic criteria have been established in the setting of stroke: the persistence of a MEP at the acute phase of stroke after stimulation of the injured hemisphere is a better predictor of recovery, while the lack of response, indicating a hypoexcitability, is associated with a poor functional outcome in the first study, to my knowledge, which prospectively followed stroke patients up to 1 year (Delvaux et al., 2003); feasibility studies had been performed earlier, but in those studies, patients were only followed 14 days (Rapisarda et al., 1996) and 90 days (Catano et al., 1995). But distant healthy brain regions matter too: unilateral brain injuries have a remote impact called “diaschisis”. This term introduced by von Monakow in 1914 (Finger et al., 2004) refers to the effects of a focal cerebral lesion on areas which are anatomically distant, but functionally related given the underlying connecting neural network (Carrera and Tononi, 2014). Originally, the concept was mainly clinical, but is easy to demonstrate through different functional imaging methods that show a change of the cerebral blood flow in target regions: the cerebellar diaschisis (Baron et al., 1981) and the transcallosal diaschisis on the contralateral cortical areas (Kataoka et al., 1989). The mechanism is a deafferentation i.e., the interruption of activation of the healthy target structure, from the injured cortex (or injured subcortical area). This activation can be excitatory or inhibitory, resulting in a modification of the metabolism and local blood flow, according to the neurovascular coupling principle established by Roy and Sherrington (1890). The importance of the cortico-cerebellar diaschisis observed at the acute phase of stroke has been shown to have a negative predictive value on the clinical outcome at 2 months (Takasawa et al., 2002). The inter-hemispheric pathway, passing through the corpus callosum, is rather inhibitory (Schambra et al., 2003). In the healthy brain, the interhemispheric inhibition is balanced, that is to say that none of the two hemispheres is a stronger “inhibitor” than the other. After unilateral infarct, increased cerebral blood flow of the contralateral homonymous area was evidenced with functional imaging studies and according to the neurovascular coupling paradigm reflecting thus its increased activity. Surprisingly, this was correlated to the most severe deficits (Ward and Frackowiak, 2006). This is interpreted as that the contralateral hemisphere continues to exert its inhibitory tone on the ischemic hypoactive hemisphere, thereby contributing to the worsening of the neurological deficit: the ipsilateral ischemic cortex becomes doubly impaired, by the stroke itself and by the exaggerated unbalanced inhibitory impulse from the healthy contralateral hemisphere. This imbalanced interhemispheric inhibition is evidenced as soon as the 1st week after stroke. TMS paired-pulse protocols, allows evaluation of GABA-A mediated intracortical inhibitory circuits (short-interval intracortical inhibition) and GABA-B mediated intracortical inhibitory circuits (long-interval intracortical inhibition). A longitudinal study of 10 stroke patients followed-up during 6 months used TMS to identify prognostic factors. It suggests that recovery, during the acute period, correlates to the relative integrity of the ipsilateral cortico-spinal pathway of the affected hemisphere (measured by MEP and motor threshold), whereas, after the acute phase, it correlates to the development of alternative neural networks on both hemispheres (as measured with short- and long-interval intracortical inhibition; Swayne et al., 2008).

In this small cohort, the importance of the contralateral hemisphere seemed to be more prevalent in large infarcts and less important in small infarcts. However, because of the small cohort and heterogeneous lesions (in the anterior or posterior circulation territory, cortical or subcortical), extrapolating data to all stroke patients, is therefore difficult. Yet, in animal studies, blocking the activity of the unaffected hemisphere by lidocaine application on rats with a middle cerebral artery occlusion performed 4 weeks prior to injection worsens the hemiparetic deficit, especially if large lesions are induced (Biernaskie et al., 2005). Thus the interhemispheric imbalance at the acute phase is detrimental but would participate to recovery thereafter. The constraint induced therapy, now widely used in physical medicine and rehabilitation units, is the direct implementation of these observations. The principle is a “forced” use of the paretic limb and a forced non-used of the healthy limb. This has two consequences: the corresponding contralateral primary motor cortical area to stroke is less strongly activated, limiting its inhibitory transcallosal and deleterious effect on the ischemic hemisphere and the ispilateral hemisphere is instead overactivated. In a meta-analysis of randomized studies of “constraint induced therapy”, Bonaiuti et al. (2007) concluded that there is a steady improvement of the paretic limb, yet without being able to provide a standardized program because of all the different protocols that were used and of the small groups of patients. Interestingly, this clinical improvement was correlated to the doubling of the excitability parameters of the injured hemisphere measured with TMS (Liepert et al., 2000).

## Non-Invasive Brain Stimulation

Repetitive TMS (rTMS) acts like a neurostimulator. It involves a continuous train or periodic trains of pulses that change the cortico-spinal excitability and mechanisms could be analogous to LTP or its counterpart, long term depression (LTD). The daily stimulation of the same area for about 20 min is repeated for 1 week or 2. The effect depends on the pacing rate. High frequency stimulation (i.e., >3 Hz) increases cortical excitability while a low frequency stimulation (i.e., ≤1 Hz) decreases cortical excitability.

This tool is painless and simple to use in an awake patient. The precise location of the coil over the primary motor cortex (M1) is easy to verify, since it induces an involuntary contralateral muscle contraction that can be registered as a MEP. As soon as the target-stimulated area is outside M1, a real-time neuronavigation, coupled to the own patient’s cerebral MRI, is recommended to improve the position of the coil. It requires large and expensive equipment and cannot be performed at bedside. However, spatial and temporal resolution is high (Klomjai et al., 2015).

Theta burst stimulation is a modified form of rTMS. It consists of pulses applied in bursts of three at 50 Hz with an interburst interval at 5 Hz. Intermittent theta burst stimulation involves 2 s of TBS trains repeated every 10 s and increases excitability, whereas continuous theta burst stimulation involves uninterrupted TBS trains for 20 or 40 s and decreases cortical excitability (Chung et al., 2016).

Transcranial direct current stimulation (tDCS) acts rather as a neuromodulator. It is an easier electrophysiological tool to handle, much smaller and portable at the patient’s bedside. It delivers weak polarizing direct currents to the cortex via two large electrodes placed on the scalp. The active electrode is applied over the targeted cortex area and a direct current generator (0.5–2 mA) is delivered to modify the threshold of cortical neurons and the underlying cortex excitability. It is polarity dependent: anodal stimulation increases the network excitability and cathodal stimulation decreases the network excitability (Nitsche and Paulus, 2001a,b). Moreover, because of its size, tDCS is easier to apply concomitant with a behavioral task, or during physical or occupational therapy (Roche et al., 2015).

## Non-Invasive Brain Stimulation (NIBS) in Stroke

More than 1400 publications so far involve NIBS in humans, 230 of these being devoted to stroke. They concern mostly upper limb motor function assessment and to a lesser degree speech disorders. More recently some articles treated the impact of NIBS on post-stroke aphasia, apraxia, neglect, gait and coordination impairment, but we will focus on motor deficit and NIBS. NIBS therapeutic strategies, in stroke, were developed to enhance “adaptive” plasticity and to counteract “maladaptive” plasticity.

### rTMS

Because of the higher risk of epilepsy at the acute phase, first feasibility studies were designed to test the inhibition of the contralateral non affected primary motor area, 3–12 months after the stroke onset at rTMS (Mansur et al., 2005; Takeuchi et al., 2005; Fregni et al., 2006). Patients were submitted to a protocol of a single (30 min; Mansur et al., 2005; Takeuchi et al., 2005) or repeated sessions (20–30 min a day during 5 working days (Fregni et al., 2006). One study which used excitatory rTMS on the ipsilateral affected hemisphere was then conducted in chronic strokes at 10 Hz (Kim et al., 2006), evaluated immediately after the procedure. For these first four studies, the number of included patients was relatively small (10–20). Khedr et al. (2005) showed that there was a benefit to deliver a high frequency stimulation (3 Hz) to the ipsilateral affected hemisphere at the acute phase, 10 days after the onset of stroke, in a larger cohort (*n* = 52). They showed, comparing two high-frequency stimulations, that there was no additional benefit to deliver a higher excitation (10 Hz vs. 3 Hz) of the primary cortex (Khedr et al., 2010). MEP and motor threshold were also significantly modified in the treated groups.

These studies were small (even in the larger studies, 20% of patients were lost to follow-up), but were relatively homogenous clinically and radiologically with most of the time subcortical infarcts. They were randomized in a “crossover” study to receive the real or “sham” stimulation, with a 1 week washout period, or were randomized to receive one or the other stimulation. Crossover studies have been used most of the time in patients submitted to one rTMS session and one sham session separated by 1 week. The order of the sessions was randomly assigned and the measurement consisted most of the time on assessing the strength of hand grip or pinching force and velocity (Takeuchi et al., 2005; Nowak et al., 2008). When specifically looked at, the effect of the rTMS had disappeared within 30 min, suggesting that it did not interfere with the results of the second session. These crossover studies had the advantage to require a smaller number of patients. On the contrary, randomization of patients receiving one or the other stimulation was chosen after repeated sessions. The “Sham” procedure was not always described. Variable placebo conditions have been used, such as changes of the coil orientation (Takeuchi et al., 2005; Kim et al., 2006) or of the target area (Nowak et al., 2008). “Sham coils” have been manufactured delivering a magnetic field of only 10% of the effective coil. They are supposed to deliver a persistent cutaneous somatosensory sensation, similar to the one provided by the real coil over the scalp (Fregni et al., 2006), but the sensation is not quite the same (Loo et al., 2000). In other articles, no magnetic current was delivered (Avenanti et al., 2012). In that last case, the patient did not had any sensation over the scalp, but the authors justified this placebo procedure by putting forward that the patients were naïve to rTMS and thus had no mean of comparison (used in randomized groups). This might be true if the patients included in the real arm or in the sham arm did not talk together after the session. This crucial aspect of controlled trials at rTMS has been extensively discussed previously (Lefaucheur et al., 2014). Clinical evaluation was performed using manual skills (finger tapping), neurological scores (NIH Stroke Scale (NIHSS), Scandinavian stroke scale) and/or disability scores (Barthel, Fugl-Meyer scales). These last scales have the advantage of better considering a global function and the consequence of improvement in daily living, rather than a relative improvement of finger dexterity. Improvement was seen immediately at the end of the procedure, with a long-lasting effect obtained when the stimulation was repeated 5 or 10 days (Khedr et al., 2005; Fregni et al., 2006), up to 1 year (Khedr et al., 2010). Finally, a study evaluated the benefit in 19 patients, 12 months after the stroke onset of a combination therapy of high-frequency repetitive stimulation (20 Hz) on the affected ipsilateral hemisphere and concomitant “constraint-induced therapy” during 10 working days, This study did not support the adjuvant use of rTMS to the constraint induced therapy, which does not preclude a possible improvement when performed at an earlier stage of the disease (Macolm et al., 2007).

These studies paved the way for all subsequent trials. Three meta-analyses, including controlled randomized studies and excluding studies where evaluation was only assessed by neurophysiological measurement, were controversial (Klomjai et al., 2015).

• A Cochrane database analysis of the ability of rTMS to improve motor function after stroke selected 19 randomized controlled trials and 588 patients between 2002 and 2012, regardless of the delay after stroke (4 h to 6 years), the ischemic or hemorrhagic type, the cortical or subcortical location, the initial severity, the type of evaluation, but they excluded studies where only electrophysiological parameters were assessed. The study failed to support the use of rTMS for stroke rehabilitation (Hao et al., 2013).
• Hsu et al. (2012), selected 18 studies and 392 patients between 1990 and 2011. By contrast with the Cochrane review, they showed that the effect of NIBS was more effective in improving daily living activities and motor function in subgroups of patients with subcortical infarcts, and with a protocol using low frequency rTMS over the unaffected hemisphere. One may speculate that because overlying cortex is intact, plastic capacities are preserved.
• A meta analysis on the effect of rTMS on post-stroke aphasia selected seven studies involving 160 patients, between January 1965 and October 2013. They concluded that low-frequency rTMS with a 90% resting motor threshold that targets the triangular part of the right inferior frontal gyrus has a positive effect on language recovery but that further studies with larger populations are required to assess the long-term effects of rTMS (Ren et al., 2014). Otal et al. (2015) confirmed this promising approach with cathodal tDCS or low-frequency rTMS.

These meta-analyses of very heterogeneous studies do not reflect the results of individual studies. Some points deserve a specific attention (Lüdemann-Podubecká et al., 2015): there is a more positive effect of facilitatory rTMS at the acute phase and inhibitory rTMS at the chronic phase. NIBS is more effective in case of subcortical infarcts, in mild to moderate motor hand deficit and in males (but who have stroke at an earlier age than women thus providing a bias in the interpretation of these data). When rTMS is applied over a maximum of five sessions, it provides long-lasting effects, with no further benefit when applied during 10 sessions.

### tDCS

tDCS holds particular promise because the equipment is inexpensive and easier to manipulate, comparatively to rTMS. As for rTMS, there is also evidence that repeated sessions of tDCS induce a longer duration of motor deficit recovery. In line with the paradigms used with the rTMS protocols, cathodal stimulation of the unaffected hemisphere, to inhibit abnormally high levels of interhemispheric inhibition from the contralesional M1, and anodal stimulation of the affected hemisphere, to reverse the ipsilesional hypoexcitability, have been proposed. As for rTMS, repeated sessions of tDCS, have mainly been performed at the chronic stage of the disease. Boggio et al. (2007) have shown that hand motor function, blindly evaluated with the Jebsen-Taylor Hand function test, improved after either cathodal tDCS of the unaffected hemisphere or anodal tDCS of the lesional hemisphere, when compared to sham tDCS. Moreover, there was a long lasting effect of at least 2 weeks when cathodal tDCS was performed five consecutive days. Lindenberg et al. (2010) assessed the beneficial additional bihemispheric tDCS stimulation (cathodal stimulation of the unaffected hemisphere and anodal stimulation of the affected hemisphere) of 20 chronic (>5 months) stroke patients submitted to simultaneous physical/occupational therapy. The improvement of the motor function was greater in the group treated by real stimulation (+21% in Fugl-Meyer and 19% in Wolf Motor Function test scores) compared to the sham stimulation, and lasted at least 1 week. Moreover, cerebral functional magnetic resonance imaging (fMRI) showed a stronger activation of the ipsilesional primary and premotor cortex during paced movements of the affected limb in the real stimulation group only. The same gain of function was obtained by others (Stagg et al., 2012; Goodwill et al., 2016), but in this last study, patients were separated into three groups evaluating separately the three conditions (cathodal vs. anodal vs. sham stimulation) although a lesser effect on hand motor deficit was observed (5–10%). In the same line, Rocha et al. (2016) compared these three strategies to constraint induced movement therapy, in order to reinforce the “rebalancing” of cortical excitability between the two hemispheres. This trial was double-blind sham controlled and upper limb motor recovery was assessed with the Fugl-Meyer assessment, the motor activity log scale and the handgrip strength at the end of the 4 weeks session, the tDCS being performed three times a week. The anodal tDCS only achieved an improvement in the Fugl-Meyer assessment score. Altogether, the ipsilesional anodal tDCS seems to have a greater impact than contralesional cathodal tDCS and sham stimulation on motor function of chronic stroke patients. Using a paradigm of bilateral tDCS combined with upper extremity robot-assisted therapy over 2 weeks, Straudi et al. (2016) showed that the Fugl Meyer assessment score was significantly better in chronic stroke patients with a subcortical lesion than in patients in a subacute phase after stroke or with a cortical stroke.

Two recent meta-analyses could not solve the controversy of whether tDCS is effective in long term motor recovery.

• Marquez et al. (2015) selected 15 studies with 315 patients. Cortical stimulation did not statistically improve motor performance when measured immediately after the intervention whatever the anodal, cathodal or bihemispheric stimulation. However in subgroups with chronic and moderate impairments, improvement was statistically significant.
• The Cochrane review (Elsner et al., 2013) selected 15 studies with 455 patients. They could not conclude that there was a strong evidence of effectiveness of tDCS in stroke.

### Combined Approaches

Whether there is a potential effect when combining NIBS, either using rTMS or tDCS, with intensive physical therapy, constraint-induced therapy (Macolm et al., 2007), robot-therapy (Hesse et al., 2007; Triccas et al., 2015), EMG-triggered functional neuromuscular stimulation (Theilig et al., 2011) is controversial and could not be evidenced in recent studies. The reason of this failure is unclear. The first hypothesis is that there might be a ceiling effect obtained after the first procedure. The second hypothesis is that instead of a priming effect induced by the first procedure, on the contrary, an inhibitory effect of the adjuvant therapy is produced. This should be understood in the light of what is known about metaplasticity, that is the plasticity of plasticity, meaning that activity-dependent synaptic plasticity has been modified by prior synaptic activity, which may shift the threshold for LTP and LTD induction (Cassidy et al., 2014); or homeostatic plasticity for homeostasis of plasticity, which provides a means for neurons and circuits to maintain functions stable in the face of synaptic perturbations (Bolognini et al., 2009). In that respect, NIBS, according to the moment it is applied (before, during, or after neurorehabilitation), could interact with the motor task and have opposite and invalidating effects. The next step should aim at better understanding of the interaction of motor training and NIBS according to the moment it is provided (Kakuda et al., 2012) since it could be an additional variable that influences the synaptic state.

## Experimental Data

What cellular and molecular mechanisms underlie those complex mechanisms and are able to take over the effects of the electrophysiological process is unclear and poorly explored. In a model of experimental stroke, induced by transient 90 min of middle cerebral artery occlusion, rats were exposed 4 days later to a 1 Hz, 5 Hz, and continuous or intermittent theta-burst rTMS protocol. At the end of the stimulation period (Ljubisavljevic et al., 2015), only intermittent theta-burst rTMS protocol induced differential up-regulation of 52 genes in rat cortices, these genes being involved in angiogenesis, inflammation, neuroprotection and cellular repair, all these mechanisms promoting recovery (Nih et al., 2012; Poittevin et al., 2013). In the same model, but with a different rTMS protocol using high-frequency stimulation applied 1 h after the onset of ischemia, neuroprotective effect was evidenced via anti-apoptosis of the cells located at the margin of the infarct (Gao et al., 2010). This result was confirmed after 10 Hz stimulation applied over a 2 week period that showed an up-regulation of Bcl2- and a down-regulation of bax- positive cells, together with an improved neurological deficit (Yoon et al., 2011). Optimal parameters were also investigated in the same model of experimental stroke. When applying diverse electrical protocols of stimulation (3–7 days, 0–200 μA, 0–50 Hz), over the ischemic boundary in the epidural space, best efficacy was obtained at 2 Hz and 100 μA: infarct volume was reduced, neurological deficit and cerebral blood flow were improved, angiogenesis was increased and inflammatory cells were diminished. Moreover, after 1 week of electrical stimulation, BDNF, glial cell-derived neurotrophic factor (GDNF) and vascular endothelial growth factor (VEGF) were up-regulated in the ischemic penumbra of the treated animals (Baba et al., 2009).

## Predictive Factors of Recovery and How to Design Future Trials?

Both rTMS and tDCS induce long-term effects, whose magnitude of improvement ranges between 10 and 20% (Talelli and Rothwell, 2006), according to the upper limb motor functional assessment that have been used in the literature, and after at least five sessions. High-frequency stimulation on the ispilateral affected hemisphere at the acute phase (6–29 days) is more effective than low-frequency stimulation on the contralateral non affected hemisphere (Sasaki et al., 2013).

Controlled randomized series are usually small and monocentric, and it is therefore very difficult to propose a predictable pattern of response to NIBS in stroke. Knowing what enhances or dampens rTMS effects could help to improve our therapeutic protocols. Many factors have to be considered that each may influence the stroke outcome. Five fields of action could be identified so far.

The first point is related to autorepair mechanism involved in the first weeks/months after stroke that can contribute to biased conclusions in NIBS studies. Intrinsic post-stroke recovery is dependent on disinhibition of redundant neural circuits (the rubral pathway for the descendant lateral corticospinal pathway for instance), besides the formation of new neural networks (Rossini et al., 2007). fMRI focusing on chronic strokes has shown the contribution of other indirect motor pathways (reticulospinal, rubrospinal tracts) in upper limb motor recovery (Bradnam et al., 2012; Lindenberg et al., 2012; Jang et al., 2013). Baseline clinical evaluation alone has poor prognosis accuracy on the stroke outcome at 6 months (Gorelick, 2012). The MEP measured after TMS is more promising since 30% of the patients who have a complete deficit of the upper limb still have a MEP, although not fully satisfactory due to its poor negative predictive value (Pizzi et al., 2009; Di Lazzaro et al., 2010 ; Stinear et al., 2014). On MRI, the mean fractional anisotropy value between both sides on the posterior limb of the internal capsules values, a structural marker of descending motor corticospinal tracts integrity, at day 30 in the ipsilateral and contralateral corticospinal tract in the pons, is an independent predictor factor of motor outcome after 2 years (Puig et al., 2013). An algorithm has been proposed to identify key predictors of post-stroke motor recovery. It combines clinical “SAFE score”, neurophysiological (presence of a MEP at the first week) and imaging (mean value of fractional anisotropy) parameters (Stinear et al., 2012). To identify accurate prognostic factors is essential to stratify patients in clinical cohorts, and if possible in order to establish the modulation value of NIBS on stroke. Moreover, the mean age of the patients of these cohorts was 58.46 years old (Crosson et al., 2015), which is lower than the mean age of the stroke population (75% are over 65 years old; World Health Organization (WHO)). Because these factors may interfere and bias the final response, there is a need for pre-randomization stratification based on prognostic factors using if possible the predicting recovery potential (PREP) algorithm which combines clinical, electrophysiological and imaging criteria. Moreover, if one looks at individual variability in stroke recovery, it might be that we have to cope with interindividual plasticity potential among patients. The genetic polymorphism of BDNF val66met induces a diminished secretion of BDNF, which is strongly involved in synapse plasticity (Cheeran et al., 2008). Also the polymorphism within the 5-HT_1A_ receptor C/C is more susceptible to respond to TMS therapy than C/G and G/G polymorphism (Zanardi et al., 2007), as are subjects affected by missense mutation of the GABAA receptor, GABRG2 (R43Q; Fedi et al., 2008). Therefore, to identify potential responders it will be essential to strengthen future clinical trials: the BDNF polymorphism, the cerebral vasoreactivity observed with functional imaging (Lang et al., 2005; Stagg et al., 2009; Stagg and Johansen-Berg, 2013; Sallustio et al., 2010) or even proton magnetic resonance spectroscopy (^1^H-MRS) which allows quantification of brain metabolites such as GABA and glutamate in a region-specific manner (Tremblay et al., 2014). Hence, rTMS according to the meta-analyses and systematic reviews of the literature published so far, is less efficient if the stroke is initially clinically more severe, if integrity of the corticospinal tract is lost, if the infarct is cortical, and of course, depends on the protocol chosen, according to the time course of the infarct. Because stroke is an evolutionary disease, with a different time course between individuals, patients should be stratified in the studies according to the factors that have been identified so far to produce good or bad recovery, using the PREP algorithm for instance, or genetic polymorphisms.

The second point concerns the ideal stimulated brain area. Although the targeted region of stimulation has always been the primary cortex area, stimulating rather premotor areas present many advantages (Plow et al., 2015). Premotor areas and supplementary motor area (SMA) are spared during the middle cerebral artery occlusion (the most frequently encountered in stroke patients), and imaging studies have shown that there is a remapping towards these areas in the long-term. The influence of premotor cortex of the unaffected hemisphere on the M1 affected hemisphere is excitatory in case of large lesions and inhibitory in case of small lesions, as shown using fMRI and TMS techniques (Bestmann et al., 2010), and could represent an alternative strategy in case M1 is injured. Moreover, there is an expansion of the representation of the distal representation of the paretic forelimb into the SMA at 3 months in the squirrel monkey, which is proportionate to the size of the M1 lesion (Eisner-Janowicz et al., 2008). At last, SMA is implicated in bimanual complex movements and into programming of these complex movements, which is an important point to address in motor skill recovery. Future trials in stroke should aim to concomitantly evaluate different targets areas as having done in dystonia (Kranz et al., 2009).

The third point concerns the stimulation parameters. The NIBS protocol might possibly also have a role in the optimal response: frequency, intensity, orientation of the coil, pattern, number of pulses by train, total number of pulses, number of trains and interstimulus interval, duration of stimulation, frequency and intensity of stimulation, number of sessions.

The fourth point would be related to the clinical research itself and better designed studies are an essential point with double blind control randomized studies and upper limb motor functional scores as an outcome measure. Indeed, the outcome measures are also very heterogeneous between studies, from measuring the reaction time at a brief motor task such as finger tapping, to scoring motor deficit or limb function (the NIHSS score, the Scandinavian stroke scale, the Fugl-Meyer scale) or more general daily-life disability scales (Rankin or Barthel). The delay of evaluation after the end of stimulation is also extremely different (immediately after the train of impulses or 3 weeks to 1 year post-stimulation). Few of the studies had also a neuroimaging/cerebral blood flow or electrophysiological supplementary measure to strengthen their clinical results and are not often able to correlate the outcome measures to the electrophysiological procedure.

The fifth and last point questions the synergistic impact of adjuvant therapy such as the intensive motor training, constraint induced therapy and robot assisted therapy. When it must be given to the patient is not that simple to solve and has been discussed above. The influence of drugs (the sodium channel blocker carbamazepine; the calcium channel blocker flunarizine, the NMDA antagonist receptor dextromethorphane) on cortical excitability changes elicited by tDCS has been demonstrated (Bolognini et al., 2009) and they should appear clearly in all studies.

## Safety Issues

The most frequently reported adverse events are not specific to the stroke population: mild headache (2.4%), anxiety (0.3%), neurocardiogenic syncope after initial exposure to rTMS (0.6%), exacerbation of pre-existing insomnia (0.3%) and local discomfort at the site of the stimulation (Hao et al., 2013). In children and adolescents, adverse events are roughly similar to what is observed in adults: after tDCS protocols, headache (11.5%), scalp discomfort (2.5%), twitching (1.2%), mood changes (1.2%), fatigue (0.9%), tinnitus (0.6%), tingling (11.5%), itching (5.8%), redness (4.7%) and scalp discomfort (3.1%) have been reported (Krishnan et al., 2015).

The only potential serious adverse side effect is seizures (Rossi et al., 2009). Some contributing factors have been isolated from the general experience of NIBS (besides stroke particularity). In epileptic patients, NIBS may be complicated by an epileptic seizure during stimulation in 0–3.6%, but it does not interfere with the course of the disease. The probability is increased if the plasma concentration of the anti-epileptic is low, if there is a high frequency of interictal epileptiform discharges (>10/min), in case of frequent complex temporal seizures (>4/month); if stimulation has been preceded by a recent epileptic seizure (<48 h) and if the epileptogenic area is specifically stimulated. In non-epileptic patients, there is a higher risk to induce an epileptic seizure if there is a familial history of epilepsy, if the patients receive regular epileptogenic psychotropes, if there is a chronic abuse of alcohol or cocaine, an underlying neurological disease, a severe heart disease, sleep disorders, if young age, female sex, and according to the characteristics of the stimulation parameters (high-frequency stimulation, long lasting train of impulses, short intertrain interval, motor area stimulation; Rossi et al., 2009; Chervyakov et al., 2015).

Stroke patients represent a population at risk, since 3–30% of the patients will develop epilepsy with a maximal risk during the first year of follow-up, depending on the lesion size and location, and on genetic and environmental factors (Pitkanen et al., 2015). However, this complication has been rarely reported in the general literature of NIBS whatever be the underlying disease: 16 cases in adults in the safety guidelines of the use of rTMS (Rossi et al., 2009), and two cases in children/adolescents (0.67%) treated for a severe depression (Krishnan et al., 2015). However, in stroke, only small cohorts were investigated with a limited number of stimulations, and even less at the acute stage. This should prompt investigators to respect safety recommendations.

An interesting question is the risk taken for the operator; although there are no specific data, the exposure for the magnetic field pulses is considered limited when the operator stays at more than 0.7 m from the surface of the coil (Rossi et al., 2009).

## Concluding Remarks

Both rTMS and tDCS induce after effects, whose magnitude of improvement ranges between 10 and 20% (Talelli and Rothwell, 2006), according to the upper limb motor functional assessment that have been used in the literature. Tolerance is excellent. The meta-analyses conclusions do not match with those of individual clinical trials, but heterogeneity of the stroke history, the individual susceptibility, the outcome measures, and the lack of fundamental knowledge on the place to give to adjuvant therapies and the influence of concomitant drugs complicate interpretation. At last, the question whether there is a therapeutic window for synaptic plasticity remains unsolved. It is illusory to think that rehabilitation strategies can be optimally designed without understanding all the mechanisms that are invested and their time course after stroke. Combining approaches enhancing adaptive plasticity and limiting maladaptive plasticity according to the stage of the disease, pharmacological, electrophysiological or physical adjuvant therapy could theoretically improve the patients’ care, and given the disease complexity, most of all, should ultimately favor a patient-tailored approach.

## Author Contributions

The author confirms being the sole contributor of this work and approved it for publication.

## Conflict of Interest Statement

The author declares that the research was conducted in the absence of any commercial or financial relationships that could be construed as a potential conflict of interest.
